# A Diet Supplemented with Polyphenols, Prebiotics and Omega-3 Fatty Acids Modulates the Intestinal Microbiota and Improves the Profile of Metabolites Linked with Anxiety in Dogs

**DOI:** 10.3390/biology11070976

**Published:** 2022-06-28

**Authors:** Eden Ephraim, Jeffrey A. Brockman, Dennis E. Jewell

**Affiliations:** 1 Pet Nutrition Center, Hill’s Pet Nutrition, Topeka, KS 66617, USA; jeff_brockman@hillspet.com; 2 Department of Grain Science and Industry, Kansas State University, Manhattan, KS 66506, USA; djewell@ksu.edu

**Keywords:** anxiety, 4-ethylphenyl sulfate, metabolome, microbiota, gut-brain axis

## Abstract

**Simple Summary:**

This study used a nutrition-based approach to examine the effects of foods supplemented with fish oil and a polyphenol blend (citrus pulp, carrot, and spinach) with or without added tomato pomace on anxiety-related biomarkers in dogs. First, all dogs consumed the same initial food, then either the control or test (with tomato pomace) foods, then the washout food, then switched over to the test or control foods, each for 30-day periods. Many more changes in plasma and fecal metabolites were observed when comparing the washout food with the control or test foods than when the control and test foods were compared. Plasma levels of several metabolites that were previously associated with anxiety disorders, including 4-ethylphenyl sulfate, were decreased with the control or test foods compared with the washout food. In addition, bacterial genera that are decreased in the feces of those with anxiety-like disorders were increased following the consumption of the control or test foods. Overall, these data indicate that foods supplemented with omega-3 fatty acids and selected fiber and polyphenol sources lead to beneficial changes in anxiety-related metabolites and gut bacteria.

**Abstract:**

A nutrition-based approach was utilized to examine the effects of fish oil and a polyphenol blend (with or without tomato pomace) on the fecal microbiota and plasma/fecal metabolomes. Forty dogs, aged 5–14 years, were fed a washout food, then randomized to consume a control (fish oil and polyphenol blend without tomato pomace) or test (fish oil and polyphenol blend with tomato pomace) food, then the washout food, and crossed over to consume the test or control food; each for 30 days. Several metabolites differed when comparing consumption of the washout with either the control or test foods, but few changed significantly between the test and control foods. Plasma levels of 4-ethylphenyl sulfate (4-EPS), a metabolite associated with anxiety disorders, demonstrated the largest decrease between the washout food and the control/test foods. Plasma 4-EPS levels were also significantly lower after dogs ate the test food compared with the control food. Other plasma metabolites linked with anxiety disorders were decreased following consumption of the control/test foods. Significant increases in *Blautia*, *Parabacteroides*, and *Odoribacter* in the fecal microbiota correlated with decreases in 4-EPS when dogs ate the control/test foods. These data indicate that foods supplemented with polyphenols and omega-3 fatty acids can modulate the gut microbiota to improve the profile of anxiety-linked metabolites.

## 1. Introduction

The gut-brain axis involves a communication loop among the brain, gut, and gut microbiota [1,2]. Gut microbiota can metabolize food from the host into a variety of metabolites that then may enter host circulation and influence the central nervous system, while the brain may affect the gut microbiota by influencing gut motility, secretion, and permeability [1,2].

Anxiety disorders are quite prevalent in human beings as well as in dogs [3,4], with about 26–29% of dogs exhibiting fearfulness, an anxiety-related disorder that increases with age [5,6]. In addition, some dogs may exhibit behavioral phenotypes (canine dysfunctional behavior) similar to autism spectrum disorders (ASD) in human beings, including repetitive motions such as tail chasing as well as trance-like behavior [7,8]. Some work has been done to characterize the microbiome in people and dogs with anxiety or ASD [9,10,11,12]. Several studies have shown that transplantation of fecal microbiota from patients with a variety of psychiatric conditions into germ-free mice appears to result in the development of these conditions in the mice as measured by behavioral and physiological parameters. A systematic review and meta-analysis found that several psychiatric disorders, including anxiety, are characterized by an enrichment of pro-inflammatory gut microbiota and a reduction of anti-inflammatory genera [9]. Further, microbial metabolic pathways appear to be altered in several psychiatric and neurological disorders [13].

In addition to defining the gut microbiota in anxiety-related disorders, several studies have attempted to identify biomarkers to differentiate between subjects with anxiety and healthy controls in mice, dogs, and human beings [14]. A variety of biomarkers have been identified in anxiety disorders, including neurotransmitters, neuropeptides, neurotrophic factors, and immunological factors, but none were specific or sufficient for diagnosis [15]. One study proposed a panel of four urinary biomarkers that could distinguish patients with depression or anxiety from healthy controls, but this was based on a relatively small sample size of 16 people per group [16]. In dogs that exhibit fearfulness, greater plasma levels of glutamine and gamma-glutamylglutamine were observed compared with non-fearful dogs [3]. More recently, administration of 4-ethylphenyl sulfate (4-EPS), a metabolite produced by the gut microbiota [17], to wild-type mice induced anxiety-like behavior [18]. 4-EPS was also shown to enter the brain and impair oligodendrocyte maturation in mice and to decrease interactions between oligodendrocytes and neurons in ex vivo brain cultures, indicating a direct role in anxiety [18]. Notably, 4-EPS was 46-fold higher in serum from a mouse model of ASD compared with wild-type mice [17]. Levels of 4-EPS were restored following administration of *Bacillus fragilis*, establishing a gut-brain connection [17]. 4-EPS was also identified as one of several plasma metabolites that could most distinguish between children with ASD and those that are typically developing [19].

Part of the ability of gut microbiota to produce certain metabolites depends on the food source from the host. The influence of food on the gut microbiota and behavior has been reported in mouse studies [20,21]. However, some of the findings in rodent models have not translated well to human beings [22], though preliminary work indicates that there are links among the diet, gut microbiota, and depression/anxiety disorders [23] and mood [24] in people. Furthermore, the gut microbiota are similar between human beings and dogs [25], so studies in dogs are likely to be relevant to human beings.

Dietary polyphenols are known to confer many beneficial health effects, including amelioration of inflammation, oxidative stress, and neurodegeneration [26,27]. In particular, citrus fruits [28,29], spinach [30,31], carrots [32], and tomatoes [33,34] serve as excellent sources of polyphenols and prebiotics. In addition, tomato juice was shown to reduce anxiolytic activity in mice [35], and tomato consumption confers numerous other beneficial health effects [36]. Thus, it was hypothesized that a nutrition-based approach including polyphenols could affect the gut-brain axis via modulation of the canine gut microbiome and/or by affecting the levels of anxiety-related metabolites. While fish oil and its omega-3 fatty acids are known to confer health benefits, there are conflicting reports on their effects on anxiety [37,38]. This study aimed to evaluate effects of polyphenols and fish oil on plasma and fecal levels of metabolites and on the fecal microbiota in healthy older dogs, and to examine if any were related to anxiety. 

## 2. Materials and Methods

### 2.1. Study Foods

Three food formulations were used in this study: a washout, control, and test food, which were all manufactured at the Hill’s experimental food laboratory and met the maintenance nutrition requirements of the Association of American Feed Control Officials (2021). The washout food (to allow for standardization of all dogs on the same food) contained >10-fold more corn compared with the control and test foods. It also contained chicken meal, rice, pork, corn gluten, soybean oil, carnitine, and about half the amount of flax seed in the control or test foods. The control and test foods had rice, pea flour, chicken, barley, oat groats, egg, fish oil, lipoic acid, and a polyphenol blend of citrus pulp, carrot, and spinach. The control and test foods both contained fish oil (0.5%) and were predicted to contain about 106 mg/g more polyphenols than the washout food. The test food also contained tomato pomace in the blend as a source of polyphenols, with lycopene estimated at 0.054 ppm. 

Proximate analyses were performed using Association of Official Analytical Chemists methods as previously described [39]. Digestibility analyses were carried out as previously described [40,41], using the following equations:

True protein digestibility = [(protein intake − (fecal protein − endogenous metabolic protein))/protein intake]

Apparent protein digestibility = [(protein intake − fecal protein)/protein intake]

### 2.2. Animals and Experimental Design

Forty older healthy beagle dogs, all spayed or neutered, between 5–14 years of age, were included in the study. Dogs with compromised health conditions such as kidney disease, inflammatory bowel disease, colitis, or food allergy, and dogs that received antibiotics within the month before study start were excluded from this study. Dogs were to be removed from the study if it was to their benefit in the opinion of the colony veterinarian. All dogs were owned by Hill’s Pet Nutrition and were housed in pairs at the Pet Nutrition Center, where they had regular access to natural light, socialization with other dogs, and daily exercise.

**Figure 1:**
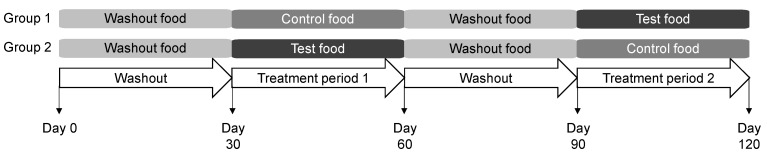
Study design in which dogs consumed the washout, control, and test foods. Each group contained 20 dogs.

The study protocol was approved by the Hill’s Institutional Animal Care and Use Committee (CP798.0.0.0-A-C-D-ADH-MULTI-120-LFS) and Animal Welfare Committee. Dogs were fed the washout food for the initial 30 days of the study and were then randomized to consume the control or test foods for the next 30 days (Figure 1). Dogs were then fed the washout food for 30 days prior to a cross-over to consume the control or test food for 30 days. Feeding was administered once daily from an electronic feeder, with the offered amount calculated for body weight maintenance. 

### 2.3. Sample Collection, Scoring, Processing, and Metabolite Analysis

Blood and fecal samples were collected at the end of each 30-day feeding period. Stools were collected within 30 min of defecation and scored from 1 to 5 as previously described, where a score of 1 indicates >75% liquid and 5 is >80% firm [41]. Feces were then homogenized in a Thinky Mixer (Thinky USA, Inc., Laguna Hills, CA, USA), aliquoted into vials, and frozen at −80 °C until further processed. 

Blood chemistry parameters were analyzed using enzymatic colorimetric methods as in a prior report [42]. Global plasma and fecal metabolite analyses were performed by Metabolon (Morrisville, NC, USA) as previously described [43,44]. Briefly, gas chromatography (for hydrophobic molecules) and liquid chromatography mass spectrometer (for hydrophilic molecules) platforms were utilized to identify and provide relative quantification of metabolites.

### 2.4. Microbiota and Bioinfomatics Processing

Total DNA was extracted from thawed fecal samples using the Qiagen MagAttract Power Microbiome DNA/RNA EP DNA isolation kit (Qiagen, Germantown, MD, USA) with the Eppendorf epMotion 5075 TMX platform (Eppendorf AG, Hamburg, Germany). As previously described [45], PCR amplification spanned the V3-V4 hypervariable regions of the 16S rRNA gene, amplicon sequencing was performed using the Illumina (San Diego, CA, USA) library preparation protocol (15044223 Rev. A), sequences were de-multiplexed to obtain FASTQ Files, and bacterial taxa were classified using the GreenGenes reference taxonomy at the genus level. Copy numbers of the 16S genes in microbial taxa were corrected and numerical values were centered log ratio (CLR) transformed for statistical analysis.

### 2.5. Statistical Analysis

Data analyses utilized JMP Pro software (JMP, Cary, NC, USA). Relative levels of metabolomics data were log-transformed prior to statistical analysis, and mean units for metabolites were scaled to a median of 1 in order to compare fold changes between samples. Treatment effect during the treatment period, using animal ID as random designate, was compared using linear mixed modeling. Metabolites that were significantly different between foods after 30 days are shown. 

Fecal microbiome data were processed as previously reported, with CLR-transformed data compared via linear mixed modeling with animal ID as a random variable [39]. Pearson’s correlation coefficient (r) regression analyses were used for correlations between 4-EPS, metabolites, and operational taxonomic units (OTUs). Statistical significance was set at *p* ≤ 0.05. 

## 3. Results

### 3.1. Food, Study Design, and Animals

The control and test foods were similar as seen via proximate analyses, with the exception of slightly higher moisture levels in the test food (Table 1). The washout food contained higher levels of crude fiber and neutral detergent fiber compared with the control and test foods, with lower levels of omega-3 fatty acids (constituents of fish oil). 

The apparent dry matter digestibility was similar for each food (Table 1). True protein digestibilities were also similar.

Fecal analysis parameters of pH and ash were not significantly different among the study foods (Table 2). Moisture was significantly lower and ammonia significantly higher in dogs after consuming the washout food compared with either the control or test foods (Table 2).

Forty healthy beagle dogs (24 spayed females, 16 neutered males) were included in the study (Table S1). The mean ± standard deviation age at baseline was 10.2 ± 2.4 years (range 5–13.7) and mean body weight at baseline was 10.3 ± 2.0 kg (range 5.7–16.1). On average, dogs consumed 163.7, 170.7, and 166.1 g of the washout, control, and test foods per day (Table 2). There were no differences in body weight following consumption of the three foods. Stool scores were not significantly different after consumption of each food (Table 2). There were no protocol deviations or adverse events encountered during the study period. 

### 3.2. Effect of the Study Foods on Blood Chemistry and Plasma Metabolites

Blood chemistry parameters were similar with no significant differences between the control and test foods (Table 3). However, albumin, BUN, cholesterol, and calcium were significantly lower following consumption of the washout food compared with the control or test foods. In addition, chloride was significantly lower after dogs consumed the test food compared with the washout food, and potassium was significantly lower after dogs consumed the control and test foods compared with the washout food. Of note, all values were within normal colony ranges despite the significant differences. 

In plasma, the levels of a number of metabolites differed when comparing the washout food with either the control or test foods (Table 4). In contrast, few metabolites changed significantly when comparing the test and control foods. 4-EPS, several uremic toxins, and other metabolites implicated in anxiety disorders, were significantly lower with either the control food and/or test food compared with the washout food. 4-EPS and sphingomyelin (d18:2/23:1) were both significantly decreased with the test food compared with the control food.

**Figure 2:**
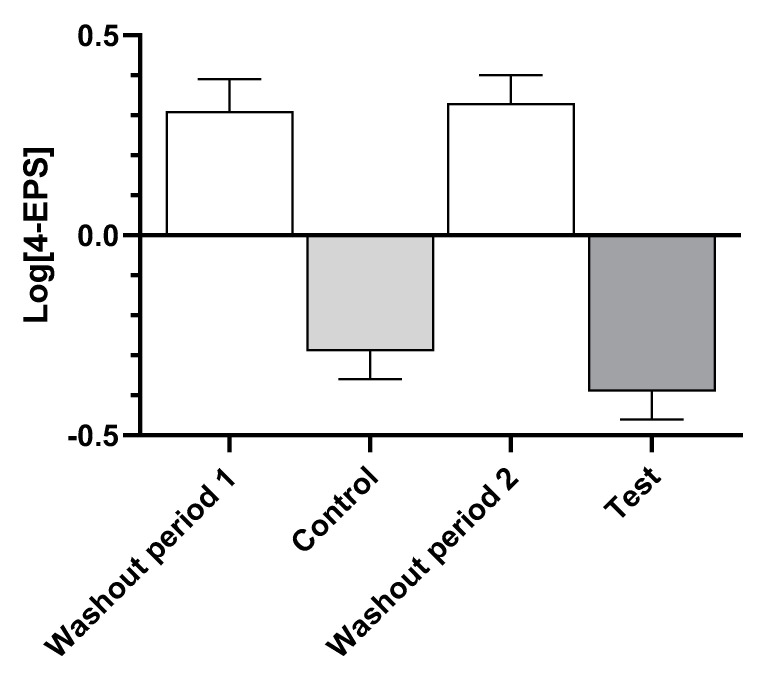
Log-transformed mean ± standard error of levels of serum 4-ethylphenyl sulfate (4-EPS) after dogs consumed the washout, control, and test foods.

Examination of the levels of serum 4-EPS (Figure 2) show that consumption of the washout food in both time periods led to increases, while both the control and test foods resulted in decreases, indicating that there was no time effect.

### 3.3. Effect of the Study Foods on Fecal Metabolites

Similar to the results with plasma, the levels of few fecal metabolites were significantly different between the control and test foods (Table 5). Levels of azelate and choline were significantly greater after dogs consumed the test food compared with the control food. Of note, 2-aminobutyrate, 4-EPS, citrate, gamma-glutamylglutamate, and sphingomyelin (d18:2/23:1) were not detected in feces.

### 3.4. Effect of the Study Foods on Fecal Microbiota

As with the plasma and fecal metabolites, many more significant differences in OTUs were observed in feces from dogs following consumption of the washout food compared to the control or test foods (Table 6). OTUs from the genera *Blautia*, *Parabacteroides*, and *Odoribacter* were among those most greatly increased following consumption of the control or test foods compared with the washout food. 

Significantly higher levels of OTUs of *Eubacterium biforme,* the genus *Acholeplasma,* and the family Coxiellaceae were seen in fecal samples from dogs fed the test food compared with the control food. Conversely, significantly higher levels of OTUs in *Mitsuaria chitosanitabida,* the genera *Flexispira* and *Anaerovorax,* the family Gemellaceae, and the class Gammaproteobacteria were seen in fecal samples from dogs fed the control food compared with the test food.

### 3.5. Correlations of 4-Ethylphenyl Sulfate with Plasma and Fecal Metabolites and OTUs

Because 4-EPS is the metabolite that showed the greatest differences in plasma levels between dogs fed the control and washout foods as well as between the test and washout foods, and it was recently shown to induce anxiety-like behavior in mice [18], correlation analyses were carried out with metabolites and OTUs. Plasma levels of 4-EPS positively correlated with levels of several known renal metabolites in plasma such as indolelactate, kynurenine, N-acetylkynurenine, and phenol sulfate (Table 7). Levels of 4-EPS in plasma showed negative correlations with levels of several metabolites known to decrease in anxiety-like disorders or depression, such as 2-aminobutyrate, DHA, hippurate, and sphingomyelin (d18:1/24:1, d18:2/24:0). In contrast to the plasma metabolites, levels of plasma 4-EPS negatively correlated with levels of several renal metabolites in feces. 

Several OTUs were significantly correlated with levels of plasma 4-EPS (Table 8). *Odoribacter*, *Blautia*, and *Parabacteroides* were all negatively correlated with plasma 4-EPS levels.

## 4. Discussion

This study aimed to investigate changes in the plasma and fecal metabolomes and fecal microbiota in senior dogs following consumption of a control food without tomato pomace or a test food containing tomato pomace. However, a limited effect of addition of the tomato pomace was observed compared with the control food, with significant changes only in the metabolites 4-EPS and sphingomyelin (d18:2/23:1) in plasma, and azelate and choline in feces. In contrast, many changes in microbiota and metabolites were observed when comparing metabolites following consumption of the washout food compared with the control or test foods. These effects are likely due to the presence of polyphenols in the fiber blend and increased omega-3 fatty acids from the fish oil in the control and test foods compared with the washout food [46]. 

Levels of 4-EPS demonstrated the largest decrease in plasma and feces following consumption of the control or test foods. A metabolite of the gut microbiome [17], 4-EPS is associated with anxiety disorders and ASD. As mentioned above, 4-EPS induced anxiety-like behavior in mice and impaired oligodendrocyte-neuron interactions [18] was at much higher levels in serum in a mouse model of ASD than wild-type mice [17], and was one of the plasma metabolites that could reliably distinguish between children with ASD and those that are typically developing [19]. Interestingly, ASD is often accompanied by gastrointestinal comorbidities including constipation [47].

A number of metabolites that changed in response to the foods tested in this study are also implicated in anxiety disorders. In general, metabolites related to oxidative stress, glutamine metabolism, and neurotransmission pathways appear to be involved in anxiety disorders [14]. High anxiety in mice is associated with high levels of intracellular reactive oxygen species, and oxidative stress has been shown to lead to anxious behavior in mice [4,48]. 2-aminobutyrate, which was significantly higher in plasma following consumption of the control or test foods in this study, appears to protect against oxidative stress by increasing cellular levels of reduced glutathione [49]. In addition to oxidative stress, 2-aminobutyrate plays a role in decreasing reductive stress, as the attack of a hydrogen atom on a sulfur atom in methionine leads to formation of 2-aminobutyrate [50]. Thus, the increased plasma levels of 2-aminobutyrate observed here following consumption of the control or test foods may indicate an increased ability to withstand oxidative and reductive stress. From a clinical standpoint, levels of 2-aminobutyrate were significantly lower in plasma from older adults in Japan who exhibited depressive symptoms compared with those in the non-depressive group [51].

The lipid components of neural membranes can influence signaling and have been implicated in psychiatric disorders [52,53,54]. Plasma levels of the polyunsaturated fatty acid DHA were significantly higher following consumption of the control or test foods in this study. This result is expected given that the control and test foods contained fish oil and showed increased level of omega-3 fatty acids compared with the washout food. Previous work showed that dietary supplementation with DHA in mice reduced anxiety-related behaviors, though only in males [52]. In human beings, those with anxiety or depressive disorders exhibit lower levels of the omega-3 polyunsaturated fatty acids DHA and eicosapentaenoic acid in erythrocyte membranes and plasma [54]. In addition, two forms of sphingomyelin (d18:2/23:1 and d18:1/24:1, d18:2/24:0) were at higher levels following consumption of the control or test foods compared with the washout food in the present study. A Dutch family-based lipidomics study of 742 people found inverse correlations between the ratio of sphingomyelin 23:1/sphingomyelin 16:0 and depression/anxiety symptoms, indicating a role for sphingomyelins in these disorders [53].

Another metabolite that exhibited increased levels in plasma following consumption of the control or test foods in this study was 1-methylnicotinamide, which is known to exert anti-inflammatory, anti-thrombotic, and vasoprotective effects [55]. Both 1-methylnicotinamide and serotonin are downstream metabolites of tryptophan, and increases in nicotinamide have been shown to increase plasma levels of serotonin [56]. Indeed, plasma levels of serotonin, which can inhibit the “fight or flight” response [15], were increased after consumption of the control food compared with the washout food in this study. While about 95% of serotonin is produced by the host via enterochromaffin cells in the gut, tryptophan metabolism by the gut microbiota regulates the availability of serotonin precursors [13]. Serotonin is also thought to play a role in ASD since a gain of function mutation in the gene encoding the serotonin reuptake transporter is found in some individuals with ASD [47].

Another tryptophan metabolite, kynurenine, was found at significantly lower levels in plasma following consumption of the test food (and numerically but not significantly lower with the control food) compared with the washout food. Plasma kynurenine levels were found to positively correlate with anxiety scores in people [57]. In addition, kynurenine, its metabolites, and dysregulated signaling were increased in the brain and gut in a mouse model of chronic restraint stress in which mice display depression and anxiety-like behaviors [58].

Indolelactate, also a product of bacterial-mediated tryptophan metabolism [59], was increased in plasma following consumption of the control or test foods in this study. Along with 4-EPS (at lower plasma levels in typically developing children), indolelactate (at higher plasma levels in typically developing children) was the other top metabolite that allowed for discrimination between children with autism and typically developing children [19].

The changes in the levels of tryptophan metabolites in response to the foods in this study is of interest in relation to prior work on the supplementation of dog food with tryptophan. In dogs with dominance or territorial aggression, dietary supplementation with tryptophan for one week reduced these aggressive behaviors [60]. Sled dogs fed supplemental tryptophan displayed a decrease in agonistic behaviors, including teeth baring, snapping, biting, and nosing [61]. In contrast, tryptophan supplementation did not result in changes in the behavior of dogs in response to the approach of familiar or unfamiliar individuals [62].

Lactate and citrate, both of which were lower in plasma following consumption of the control or test foods, were some of the urinary metabolites that were higher in people with depression and anxiety disorders that enabled discrimination from healthy controls [16]. Further, lactate derived from the gut microbiome promoted anxiety-like behaviors in a mouse model [63]. Thus, decreased levels of these metabolites may indicate benefits in anxiety.

In addition to its well-known role as a building block of proteins, glutamine is a precursor to the neurotransmitters glutamate and GABA [64] and can enhance intestinal barrier function, of key importance in the gut-brain axis [65]. Glutamate is also a precursor to the antioxidant glutathione [66]. Impairment of the glutamine/glutamate cycle is implicated in the pathology of several conditions involving the brain [64]. In this study, levels of glutamine and glutamate were both significantly lower in plasma and feces following consumption of the control or test foods compared with the washout food. Several prior studies have demonstrated that glutamine and glutamate are implicated in anxiety-like disorders and in ASD. Increased plasma glutamine, along with gamma-glutamyl glutamine, was identified as a metabolic feature in dogs that exhibited fearfulness, an anxiety-related behavioral problem [3]. That study also found that SDMA was associated with fearfulness in one of the studied breeds, Great Danes, but not in the other (German shepherds). In addition to 4-EPS, levels of glutamine were higher in serum from a mouse model of ASD compared with untreated mice [17]. Glutamine was correlated with neuroticism and trait anxiety, with increased glutamine concentrations in people with anxiety disorders [67]. Both glutamate and glutamine levels were significantly higher in plasma from children with ASD compared with healthy controls [68]. Similarly, serum glutamate levels were significantly higher in adults with autism compared with healthy controls [69].

Intriguingly, 4-EPS is a derivative of tyrosine with a very similar structure to that of the uremic toxin p-cresol sulfate [17]. 4-EPS was identified as a uremic solute in a mouse model of chronic renal failure [70] and was about 17-fold higher in plasma from patients on hemodialysis compared with healthy controls [71]. In addition to 4-EPS, several of the other metabolites whose levels showed differential effects with the control/test foods and washout food are known to be uremic toxins. Carboxyethyl-GABA, choline, dimethylarginine (ADMA + SDMA), kynurenine, and phenol sulfate were all significantly lower in plasma following consumption of the control or test foods compared with the washout food, and all were found to be higher in plasma from patients on hemodialysis [71] and/or patients with end-stage renal disease [72]. Serum SDMA can be used as a biomarker for early kidney dysfunction in dogs [73]. Levels of several of these metabolites (carboxyethyl-GABA, dimethylarginine [ADMA + SDMA], and kynurenine) are higher in feces following consumption of the control or test foods compared with the washout food, indicating that this may be a mode for their elimination. 

In addition to characterizing the plasma and fecal metabolomes in response to the study foods, the fecal microbiota was also evaluated in this study. While fecal bacterial profiles did not match those of greater abundance in human beings with end-stage renal disease [74], anxiety/depression [75,76], or ASD [77], this may be due to the absence of these conditions in the dogs in this study. Other studies have observed greater changes in microbiota in disease states than in nutritional interventions, some with notable metabolomic changes but little to no change in the microbiota [78,79].

The three genera that increased the most after dogs were fed the control or test food compared to the washout food were *Blautia*, *Parabacteroides*, and *Odoribacter*. The abundances of all of these negatively correlated with 4-EPS. Significantly lower abundances of *Blautia* were found in the gut microbiota of people with high trait anxiety compared with healthy controls [10]. Because *Blautia* were significantly increased when dogs consumed the control/test foods in this study, they may shift the gut microbiome toward one associated with a less anxious state. In addition, *Blautia* was of lower relative abundance in the gut microbiota of children with ASD compared with controls in a systematic review [80] and meta-analysis [81]. There is some conflicting evidence on *Parabacteroides* in the gut microbiota of children with ASD, as one systematic review and meta-analysis found lower percentages of *Parabacteroides* (as well as other genera) compared with controls [82], while another found higher relative abundances [81], even though the analyses utilized some of the same studies. Nevertheless, significantly reduced relative abundances of *Parabacteroides* were seen in a mouse model of chronic restraint stress, fecal abundance of *Parabacteroides* positively correlated with serotonin levels and negatively correlated with kynurenine levels in the prefrontal cortex, and administration of *Parabacteroides distasonis* partially mitigated the depression and anxiety-like behaviors in these mice [58]. Regarding *Odoribacter*, a lower relative abundance was observed in the gut microbiota of people with obsessive compulsive disorder compared with healthy controls [83], and a potential anti-inflammatory effect of *Odoribacter splanchnicus* on the gut epithelium was recently identified [84]. 

In contrast to the above genera, an OTU in the family Pseudomonadaceae was significantly reduced after dogs consumed the test and the control foods and positively correlated with 4-EPS levels. The abundance of bacteria in this family were reported to increase with aging in the bladder and seminal microbiota of human beings [85]. Future research should investigate the association of these bacteria with behavioral disorders like anxiety. 

In addition to their differential abundances in individuals with anxiety/ASD compared with healthy controls, there may be a role for these genera in short-chain fatty acid (SCFA)-mediated effects in these disorders. Digestion of tomato pomace in an in vitro model with gut bacteria led to an increase in the concentrations of SCFAs [34]. Both Blautia and *Odoribacter* produce SCFAs via fermentation of dietary fiber [84,86], while the abundance of *Parabacteroides* was correlated with SCFA concentrations in the quail intestinal tract [87]. Future work could examine the role of SCFAs and these gut bacteria in anxiety and other disorders such as ASD. Indeed, SCFAs are decreased in patients with major depressive disorder [86], have been shown to ameliorate stress-induced alterations in the brain-gut axis in a mouse model [88], and along with butyrate-producing bacteria, are at lower levels in feces of children with ASD [89].

Although the greatest number of differences in metabolites were observed when the control or test foods were compared with the washout food, there were a few key differences between the control and test foods. Notably, the only ingredient that differed between the control and test foods was tomato pomace in the test food. While 4-EPS was lower in plasma following consumption of either the control or test foods compared with the washout food, its levels were lowest following the test food. Azelate was higher in feces following consumption of the test food compared with the control food. It has anti-inflammatory properties and as such, its conjugate acid form, azelaic acid, is a first-line treatment for acne vulgaris [90]. Azelaic acid was also found to be one of four urinary metabolites that could distinguish between patients with depression or anxiety disorders from healthy controls [16]. Choline has many essential functions, from serving as a component of cell membranes as well as a precursor to the neurotransmitter acetylcholine and to the universal methyl donor S-adenosylmethionine. In this study, fecal choline levels in feces were higher following consumption of the test food compared with the control food. Dietary supplementation with choline has been shown to decrease anxiety-like behavior [91] and autism-like repetitive behavior and anxiety [92] in mouse models.

Some of the microbiota also differed significantly following consumption of the control and test foods. *Eubacterium biforme* (also known as *Holdemanella biformis*), which was of higher abundance following the test food, produces the long-chain fatty acid 3-hydroxyoctadecaenoic acid that has anti-inflammatory effects in colitis [93]. *Flexispira* and an OTU of the class Gammaproteobacteria were both of lower abundance following consumption of the test food compared with the control food, and both positively correlated with 4-EPS levels. In contrast, *Coxiellaceae* was at higher abundance after consumption of the test food and negatively correlated with 4-EPS levels.

A limitation of this study is that only healthy dogs were included. Future work could focus on studying food-based interventions in dogs exhibiting anxiety-like behaviors. Further, although most of the markers were previously reported to be associated with anxiety-like behaviors and senior dogs are more prone to anxiety [6], changes in the behaviors of the dogs were not assessed in relation to the changes in metabolites and microbiota.

In summary, this study examined a nutrition-based approach in which the control and test foods were supplemented with polyphenols and fish oil to observe changes in the levels of plasma and fecal metabolites and the gut microbiota in senior dogs. A number of differences between the control/test foods and the washout food were observed in metabolites related to anxiety-like disorders, ASD, and uremia. Overall, consumption of polyphenols and omega-3 fatty acids appeared to shift metabolites toward a profile beneficial for the gut-brain axis and kidney health.

## Figures and Tables

**Table 1:** Proximate analysis of foods used in this study.

Analyte (%)	Washout Food	Control Food	Test Food
Ash	4.3	5.5	5.4
Crude fat	13.4	13.6	13.9
DHA	<0.01	0.08	0.06
EPA	<0.01	0.1	0.09
DPA	<0.01	0.02	0.02
Omega-3 sum	0.42	0.82	0.74
Omega-6 sum	3.9	3.3	3.3
Crude fiber	1.6	1.0	1.0
Neutral detergent fiber	7.5	3.6	3.4
Crude protein	18.8	21.5	21.4
Moisture	8.1	8.1	9.0
Apparent dry matter digestibility	84.4	87.5	84.8
True protein digestibility	87.4	92.2	91.1

DHA, docosahexaenoic acid; DPA, docosapentaenoic acid; EPA, eicosapentaenoic acid.

**Table 2:** Food intake and fecal analyses following consumption of the indicated foods.

	Washout Food	Control Food	Test Food
Food intake, g	163.7 ± 0.8	170.7 ± 1.3 *	166.1 ± 1.0 *^†^
Body weight, kg	10.4 ± 2.1	10.3 ± 2.0	10.3 ± 2.1
pH	5.8 ± 0.04	5.8 ± 0.04	5.8 ± 0.03
Ash, %	6.5 ± 0.24	6.9 ± 0.22	6.8 ± 0.18
Moisture, %	70.6 ± 0.48	72.4 ± 0.41 *	71.9 ± 0.34 *
Ammonia, mmol/g	0.040 ± 0.001	0.035 ± 0.002 *	0.030 ± 0.001 *
Stool score	4.6 ± 0.5	4.3 ± 0.6	4.4 ± 0.7

Data are presented as mean ± standard error. Results were compared using linear mixed modeling with animal ID as random designate. Stool score is rated from 1–5 [41]. * Significantly different vs. washout food (*p* < 0.05). ^†^ Significantly different vs. control food (*p* < 0.05).

**Table 3:** Blood chemistry parameters following consumption of the control and test foods.

	Colony Reference Range	Washout Food	Control Food	Test Food
Albumin, g/dL	2.9–4.0	3.4 ± 0.04	3.6 ± 0.06 *	3.6 ± 0.06 *
BUN, mg/dL	6.4–20.5	11.2 ± 0.50	12.8 ± 0.81 *	12.2 ± 0.77 *
Creatinine, mg/dL	0.41–0.82	0.68 ± 0.02	0.67 ± 0.02	0.65 ± 0.02
Cholesterol, mg/dL	133–401	196 ± 5.0	215 ± 7.5 *	219 ± 7.7 *
Sodium, mmol/L	144–150	149 ± 0.19	149 ± 0.26	149 ± 0.26
Triglycerides, mg/dL	34–429	103 ± 8.3	97.9 ± 7.7	92.7 ± 8.6
Calcium, mg/dL	9.6–11.7	10.1 ± 0.06	10.2 ± 0.07 *	10.3 ± 0.08 *
Chloride, mmol/L	105–115	111 ± 0.27	111 ± 0.34	110 ± 0.30 *
Potassium, mmol/L	4.0–5.3	4.7 ± 0.03	4.6 ± 0.05 *	4.6 ± 0.06 *

Data are presented as mean ± standard error. Treatment effect during the treatment period was compared using linear mixed modeling with animal ID as random designate. BUN, blood urea nitrogen. * Significantly different vs. washout food (*p* < 0.05).

**Table 4:** Plasma metabolites of interest.

Plasma Metabolite	Washout Food	Control Food	Test Food	*p* Value, Washout vs. Control Food	*p* Value, Washout vs. Test Food	*p* Value, Control vs. Test Food
4-ethylphenyl sulfate (4-EPS)	0.33 ± 0.07	−0.29 ± 0.07	−0.39 ± 0.07	<0.001	<0.001	0.028
DHA; 22:6n3	−0.4 ± 0.07	0.43 ± 0.05	0.37 ± 0.06	<0.001	<0.001	NS
glutamate	0.09 ± 0.04	−0.01 ± 0.05	−0.02 ± 0.04	0.001	<0.001	NS
glutamine	0.04 ± 0.01	−0.03 ± 0.02	−0.04 ± 0.01	<0.001	<0.001	NS
gamma-glutamylglutamate	−0.53 ± 0.13	−0.74 ± 0.12	−0.92 ± 0.11	NS	0.002	NS
dimethylarginine (ADMA + SDMA)	0.05 ± 0.02	−0.02 ± 0.02	−0.02 ± 0.02	<0.001	<0.001	NS
1-methylnicotinamide	−0.16 ± 0.06	0.08 ± 0.04	0.08 ± 0.06	<0.001	<0.001	NS
2-aminobutyrate	−0.08 ± 0.02	0.05 ± 0.03	0.09 ± 0.03	<0.001	<0.001	NS
lactate	0.10 ± 0.04	−0.03 ± 0.04	−0.07 ± 0.03	<0.001	<0.001	NS
choline	0.09 ± 0.02	−0.07 ± 0.03	−0.07 ± 0.03	<0.001	<0.001	NS
sphingomyelin (d18:2/23:1)	−0.19 ± 0.07	0.13 ± 0.06	0.05 ± 0.06	<0.001	<0.001	0.021
sphingomyelin (d18:1/24:1, d18:2/24:0)	−0.13 ± 0.05	0.17 ± 0.05	0.12 ± 0.05	<0.001	<0.001	NS
citrate	0.02 ± 0.02	−0.02 ± 0.02	−0.02 ± 0.02	0.009	0.019	NS
carboxyethyl-GABA	0.04 ± 0.08	−0.002 ± 0.08	−0.10 ± 0.07	NS	0.049	NS
hippurate	−0.31 ± 0.15	−0.20 ± 0.15	0.05 ± 0.15	NS	0.020	NS
indolelactate	−0.08 ± 0.08	0.07 ± 0.08	0.07 ± 0.07	0.033	NS	NS
kynurenate	−0.07 ± 0.09	0.02 ± 0.08	−0.02 ± 0.08	NS	NS	NS
kynurenine	−0.02 ± 0.05	−0.06 ± 0.05	−0.11 ± 0.05	NS	0.002	NS
N-acetylkynurenine	−0.16 ± 0.10	0.04 ± 0.08	0.01 ± 0.10	0.010	NS	NS
phenol sulfate	0.10 ± 0.11	0.02 ± 0.09	−0.18 ± 0.09	NS	0.003	NS
serotonin	−0.73 ± 0.18	−0.35 ± 0.20	−0.33 ± 0.20	0.031	NS	NS
azelate (nonanedioate; C9)	0.05 ± 0.05	0.01 ± 0.07	0.16 ± 0.07	NS	NS	NS
trimethylamine N-oxide	−0.16 ± 0.09	−0.05 ± 0.09	0.05 ± 0.08	NS	0.009	NS

Values are log-transformed means ± standard error. Metabolites that were significantly different after 30 days are shown. Treatment effect during the treatment period was compared using linear mixed modeling with animal ID as random designate. ADMA, asymmetric dimethylarginine; DHA, docosahexaenoic acid; GABA; gamma aminobutyric acid; NS, not significant; SDMA, symmetric dimethylarginine.

**Table 5:** Fecal metabolites of interest.

Fecal Metabolite	Washout Food	Control Food	Test Food	*p* Value, Washout vs. Control Food	*p* Value, Washout vs. Test Food	*p* Value, Control vs. Test Food
DHA; 22:6n3	−0.54 ± 0.13	0.43 ± 0.10	0.37 ± 0.12	<0.001	<0.001	NS
glutamate	0.11 ± 0.08	−0.28 ± 0.10	−0.32 ± 0.08	<0.001	<0.001	NS
glutamine	0.09 ± 0.07	−0.22 ± 0.08	−0.43 ± 0.10	0.001	<0.001	NS
dimethylarginine (ADMA + SDMA)	−0.01 ± 0.06	0.08 ± 0.06	0.10 ± 0.05	0.048	0.003	NS
carboxyethyl-GABA	−0.10 ± 0.05	0.03 ± 0.04	0.13 ± 0.04	0.009	<0.001	NS
choline	−0.06 ± 0.06	−0.15 ± 0.05	−0.03 ± 0.05	NS	NS	0.030
1-methylnicotinamide	0.09 ± 0.19	−0.30 ± 0.21	−0.04 ± 0.16	NS	NS	NS
lactate	0.39 ± 0.14	−0.34 ± 0.18	−0.38 ± 0.17	0.001	0.001	NS
sphingomyelin (d18:1/24:1, d18:2/24:0)	0.05 ± 0.17	−0.35 ± 0.18	−0.27 ± 0.18	0.003	0.048	NS
hippurate	−0.37 ± 0.18	−0.70 ± 0.19	−0.84 ± 0.15	NS	0.040	NS
indolelactate	0.14 ± 0.23	−0.26 ± 0.22	−0.10 ± 0.19	0.040	NS	NS
kynurenate	0.56 ± 0.26	0.17 ± 0.22	−0.08 ± 0.22	NS	0.010	NS
kynurenine	−0.21 ± 0.04	0.39 ± 0.07	0.50 ± 0.07	<0.001	<0.001	NS
N-acetylkynurenine	−0.94 ± 0.14	0.70 ± 0.15	0.85 ± 0.12	<0.001	<0.001	NS
phenol sulfate	−0.01 ± 0.20	0.21 ± 0.22	−0.04 ± 0.20	NS	NS	NS
serotonin	0.12 ± 0.09	−0.06 ± 0.07	−0.19 ± 0.08	0.030	0.002	NS
azelate (nonanedioate; C9)	−0.07 ± 0.06	−0.13 ± 0.06	0.85 ± 0.06	NS	<0.001	<0.001
trimethylamine N-oxide	−0.14 ± 0.13	0.004 ± 0.14	−0.03 ± 0.13	NS	NS	NS

Values are log-transformed means ± standard error. Metabolites that were significantly different after 30 days are shown. Treatment effect during the treatment period was compared using linear mixed modeling with animal ID as random designate. ADMA, asymmetric dimethylarginine; DHA, docosahexaenoic acid; GABA; gamma aminobutyric acid; NS, not significant; SDMA, symmetric dimethylarginine.

**Table 6:** Center-log ratio means ± standard error of operational taxonomic units (OTUs) that were significantly different between groups.

OTU	Washout Food	Control Food	Test Food	*p* Value, Washout vs. Control Food	*p* Value, Washout vs. Test Food	*p* Value, Control vs. Test Food
361186 Lachnospiraceae *Blautia* unclassified	7.34 ± 0.21	8.69 ± 0.26	8.64 ± 0.26	<0.001	<0.001	NS
689975 Porphyromonadaceae *Parabacteroides*	0.86 ± 0.19	2.18 ± 0.15	2.06 ± 0.18	<0.001	<0.001	NS
1030652 Odoribacteraceae *Odoribacter*	0.64 ± 0.27	2.1 ± 0.33	1.8 ± 0.33	<0.001	<0.001	NS
93469 Mogibacteriaceae *Anaerovorax*	−2.72 ± 0.05	−1.73 ± 0.17	−2.13 ± 0.14	<0.001	<0.001	0.026
1020403 Actinomycetaceae	−2.73 ± 0.06	−2.23 ± 0.16	−2.50 ± 0.11	0.008	0.033	NS
195865 Lachnospiraceae	6.99 ± 0.33	5.93 ± 0.31	6.24 ± 0.31	<0.001	0.008	NS
13805 Veillonellaceae *Anaerovibrio*	4.10 ± 0.29	3.13 ± 0.22	2.69 ± 0.27	0.004	<0.001	NS
1016422 Pasteurellaceae *Haemophilus parainfluenzae*	0.91 ± 0.27	1.7 ± 0.20	1.77 ± 0.20	0.002	0.002	NS
1141218 Coriobacteriaceae *Eggerthella*	−1.35 ± 0.04	−1.0 ± 0.12	−1.22 ± 0.80	0.009	NS	NS
100035 Helicobacteraceae unclassified	−0.31 ± 0.17	−0.8 ± 0.12	−0.73 ± 0.12	0.034	NS	NS
4342860 Gemellaceae unclassified	−0.79 ± 0.21	−0.04 ± 0.23	−0.53 ± 0.19	0.004	NS	0.043
112457 Pasteuriaceae	−1.07 ± 0.04	−0.86 ± 0.11	−0.95 ± 0.04	NS	0.017	NS
52166 Veillonellaceae *Megasphaera*	6.57 ± 0.22	6.03 ± 0.17	6.20 ± 0.19	0.020	0.031	NS
15257 Carnobacteriaceae *Carnobacterium viridans*	−1.71± 0.03	−1.55 ± 0.06	−1.62 ± 0.04	0.008	0.017	NS
100212 Veillonellaceae	4.65 ± 0.29	3.66 ± 0.23	4.03 ± 0.22	0.003	0.033	NS
37911 Comamonadaceae *Mitsuaria chitosanitabida*	−0.04 ± 0.27	0.05 ± 0.24	−0.57 ± 0.25	NS	0.017	0.018
104145 Rikenellaceae Blvii28	−0.53 ± 0.19	−0.06 ± 0.15	−0.27 ± 0.17	0.036	NS	NS
10001 Enterobacteriaceae unclassified	−0.68 ± 0.33	0.79 ± 0.43	0.51 ± 0.47	0.001	0.015	NS
965048 Neisseriaceae	−0.48 ± 0.21	−1.05 ± 0.19	−0.83 ± 0.25	0.004	NS	NS
945478 unclassified *	1.19 ± 0.27	1.19 ± 0.24	0.74 ± 0.26	NS	0.040	0.027
1090029 Sanguibacteraceae *Sanguibacter*	−1.30 ± 0.03	−1.10 ± 0.06	−1.21 ± 0.04	0.001	0.007	NS
351494 Glycomycetaceae *Glycomyces harbinensis*	−2.68 ± 0.09	−2.51 ± 0.11	−2.37 ± 0.18	0.008	0.035	NS
804742 Euzebyaceae *Euzebya*	−0.06 ± 0.22	−0.55 ± 0.16	−0.64 ± 0.16	0.022	0.016	NS
897625 Coxiellaceae	−1.18 ± 0.04	−1.09 ± 0.05	−0.68 ± 0.15	0.011	0.002	0.012
1000602 unclassified	−2.4 ± 0.08	−2.04 ± 0.15	−2.16 ± 0.12	0.040	NS	NS
103388 Acholeplasmataceae *Acholeplasma*	−1.84 ± 0.13	−1.94 ± 0.06	−1.55 ± 0.21	NS	NS	0.046
4361046 Campylobacteraceae *Campylobacter* unclassified	−0.77 ± 0.25	0.64 ± 0.40	1.10 ± 0.43	<0.001	<0.001	NS
1141335 Helicobacteraceae *Flexispira* unclassified	0.11 ± 0.23	0.19 ± 0.20	−0.29 ± 0.21	NS	NS	0.041
10196 Enterobacteriaceae *Brenneria*	−3.24 ± 0.08	−2.70 ± 0.15	−2.82 ± 0.15	0.001	0.009	NS
579304 Lactobacillaceae	−2.97 ± 0.05	−2.73 ± 0.12	−2.78 ± 0.10	0.017	NS	NS
146665 Erysipelotrichaceae *Eubacterium biforme*	1.65 ± 0.40	0.18 ± 0.30	1.10 ± 0.35	<0.001	NS	0.009
1000161 Pseudomonadaceae unclassified	1.76 ± 0.35	−0.43 ± 0.31	−0.44 ± 0.27	<0.001	<0.001	NS

OTU number, family, and genus are shown with species where available. Treatment effect during the treatment period was compared using linear mixed modeling with animal ID as random designate. NS, not significant; OTU, operational taxonomic unit. * Class Gammaproteobacteria.

**Table 7:** Significant correlations between levels of plasma 4-ethylphenyl sulfate and levels of plasma and fecal metabolites of interest.

	Correlations with Plasma Metabolites			Correlations with Fecal Metabolites		
Metabolite	Estimate ± SE	*p* Value	r^2^	Estimate ± SE	*p* Value	r^2^
DHA; 22:6n3	−0.37 ± 0.06	<0.001	0.16	−0.63 ± 0.13	<0.001	0.16
gamma-glutamylglutamate	−0.12 ± 0.06	0.041	0.01	-	-	-
dimethylarginine (ADMA + SDMA	-	-	-	−0.20 ± 0.06	<0.001	0.10
2-aminobutyrate	−0.62 ± 0.22	0.006	0.02	-	-	-
choline	-	-	-	−0.15 ± 0.06	0.008	0.06
sphingomyelin (d18:1/24:1, d18:2/24:0)	−0.37 ± 0.13	0.007	0.01	-	-	-
azelate (nonanedioate; C9)	-	-	-	−0.25 ± 0.1	0.010	0.06
hippurate	−0.10 ± 0.04	0.021	0.02	-	-	-
indolelactate	0.37 ± 0.09	<0.001	0.01	-	-	-
kynurenine	0.72 ± 0.14	<0.001	0.15	−0.35 ± 0.08	<0.001	0.15
N-acetylkynurenine	0.28 ± 0.07	<0.001	0.31	−1.20 ± 0.17	<0.001	0.31
phenol sulfate	0.35 ± 0.07	<0.001	0.001	-	-	-
trimethylamine N-oxide	−0.32 ± 0.08	<0.001	0.004	-	-	-

ADMA, asymmetric dimethylarginine; DHA, docosahexaenoic acid; r^2^, square of Pearson’s correlation coefficient; SDMA, symmetric dimethylarginine; SE, standard error.

**Table 8:** Correlations between plasma levels of 4-ethylphenyl sulfate and OTUs.

OTU	Estimate	*p* Value	r^2^
361186 Lachnospiraceae *Blautia*	−1.03	<0.001	0.12
689975 Porphyromonadaceae *Parabacteroides*	−0.99	<0.001	0.20
1030652 Odoribacteraceae *Odoribacter*	−1.11	0.001	0.09
93469 Mogibacteriaceae *Anaerovorax*	−0.41	0.006	0.06
1020403 Actinomycetaceae	−0.37	0.003	0.07
1016422 Pasteurellaceae *Haemophilus parainfluenzae*	−0.79	0.001	0.09
10196f Enterobacteriaceae *Brenneria*	−0.31	0.023	0.04
4361046 Campylobacteraceae *Campylobacter*	−1.10	0.006	0.06
109002 Sanguibacteraceae *Sanguibacter*	−0.13	0.009	0.06
897625 Coxiellaceae	−0.30	0.003	0.07
10001 Enterobacteriaceae unclassified	−1.10	0.013	0.05
1000161 Pseudomonadaceae unclassified	0.93	0.012	0.05
1141335 Helicobacteraceae *Flexispira*	0.67	0.002	0.08
13805 Veillonellaceae *Anaerovibrio*	0.83	0.004	0.07
146665 Erysipelotrichaceae *Eubacterium biforme*	1.03	0.006	0.06
37911 Comamonadaceae *Mitsuaria chitosanitabida*	0.60	0.026	0.04
945478 unclassified *	0.83	0.002	0.08

Operational taxonomic unit (OTU) number, family, and genus are shown except where otherwise indicated; species are also shown when available. r^2^, square of Pearson’s correlation coefficient, SE, standard error. * Class Gammaproteobacteria.

## Data Availability

The data presented in this study are available on request from the corresponding author.

## Supplementary Materials

The following are available online at https://www.mdpi.com/article/10.3390/biology11070976/s1, Table S1: Animal signalment.
