# Supplementary material for: A Diet Supplemented with Polyphenols, Prebiotics and Omega-3 Fatty Acids Modulates the Intestinal Microbiota and Improves the Profile of Metabolites Linked with Anxiety in Dogs
Source: Biology (Basel). 2022 Jun 28;11(7):976. doi: 10.3390/biology11070976 (PMC9312346; doi:10.3390/biology11070976)
Supplement: Supplementary file 1 [file biology-11-00976-s001.zip › biology-1763712-supplementary.pdf]

biology

MDPI

Table S1. Animal signalment.

	Age, years	Sex	Weight, kg
**Group 1**			
1	12.9	Spayed female	5.7
2	11.7	Neutered male	11.4
3	13.7	Spayed female	9.9
4	11.7	Neutered male	8.8
5	8.0	Neutered male	12.9
6	11.0	Spayed female	9.4
7	5.0	Neutered male	11.0
8	7.0	Spayed female	8.8
9	8.4	Spayed female	8.3
10	8.0	Neutered male	11.7
11	12.3	Spayed female	8.9
12	13.6	Spayed female	7.7
13	8.2	Neutered male	10.3
14	11.7	Neutered male	10.1
15	12.3	Spayed female	7.3
16	13.7	Spayed female	10.0
17	11.7	Spayed female	11.7
18	8.4	Spayed female	7.6
19	10.1	Spayed female	8.6
20	11.7	Spayed female	8.1
**Group 2**			
21	9.3	Spayed female	13.1
22	7.8	Neutered male	12.2
23	9.2	Spayed female	10.3
24	7.8	Neutered male	11.7
25	7.0	Spayed female	10.5
26	11.7	Spayed female	10.5
27	11.7	Neutered male	13.4
28	13.5	Spayed female	9.8
29	8.0	Neutered male	16.1
30	11.9	Neutered male	11.2
31	7.0	Spayed female	8.1
32	13.2	Spayed female	10.8
33	13.5	Spayed female	9.8
34	8.0	Neutered male	13.0
35	11.7	Spayed female	10.4
36	11.7	Neutered male	10.3
37	7.0	Spayed female	9.2
38	7.8	Neutered male	12.4
39	11.7	Neutered male	11.8
40	8.5	Spayed female	8.9
